# Disparities in Opioid Use Disorder Treatment Admissions

**DOI:** 10.36469/jheor.2020.13266

**Published:** 2020-06-22

**Authors:** Peter J. Mallow, Michael Mercado, Michael Topmiller

**Affiliations:** 1 Xavier University, Cincinnati, Ohio; 2 HealthLandscape, LLC, Cincinnati, Ohio; 3 American Academy of Family Physicians, Cincinnati, Ohio

**Keywords:** Treatment Episode Data Set: Admissions (TEDS-A), disparities, Cincinnati, opioid use disorder (OUD)

## Abstract

**Objectives:**

The Cincinnati region has been at the epicenter of the nation’s unfolding opioid epidemic. The objectives of this study were twofold: (1) to compare the Cincinnati region to the United States in length of time to obtain treatment and planned medication-assisted therapy for the treatment for opioid use disorder (OUD); and (2) to assess racial disparities within the Cincinnati region in wait time and type of treatment.

**Methods:**

The 2017 Treatment Episode Data Set: Admissions (TEDS-A) from the Substance Abuse and Mental Health Services Administration (SAMHSA) was used to identify a cohort of eligible individuals with a primary substance use of opioids, including opioid derivatives. Logistic regression models were performed to assess the differences for treatment wait time and type of planned treatment. Model covariates included patient demographics and socioeconomic characteristics. Three different models were performed to assess the influence of covariates of the outcomes.

**Results:**

There were 678 766 US and 3298 Cincinnati region individuals admitted for OUD treatment in 2017. The rate per 1000 for treatment admissions was 2.08 and 1.51 (*P* value < 0.0001) for the United States and Cincinnati, respectively. The fully saturated regression results found that the odds of Cincinnati individuals receiving planned medication-assisted therapy were 0.497 (95% CI, 0.451–0.546; *P* value < 0.001). The odds of waiting longer for treatment in Cincinnati were higher than in the United States as a whole: 2.33 (95% CI, 2.19–2.48; *P* value < 0.001). In Cincinnati, there were 3102 Caucasian, 123 African American, and 73 Other admissions. The fully saturated model results found that Caucasians and Other had an increased likelihood of receiving planned medicationassisted therapy (OR 1.89, *P* value 0.039; OR 7.07, *P* value 0.002, respectively) compared to African Americans. Within Cincinnati, there was not a statistically significant difference in the likelihood of waiting time to receive treatment by race.

**Conclusion:**

Individuals seeking treatment for OUD in Cincinnati were less likely to receive planned medication-assisted therapy and were more likely to wait longer than individuals in the United States as a whole. These results suggest that the demand for treatment is greater than the supply in Cincinnati. Within Cincinnati, there does not appear to be a racial disparity in treatment type or length of time to receive treatment for OUD.

## INTRODUCTION

Since 1999, the number of national drug overdose deaths among all ages of individuals within the United States (US) has increased substantially.^1^ The amount of reported drug overdose deaths increased from 16 849 in 1999 to 70 237 in 2017. Of those accumulated overdose deaths, 47 600 reported national drug overdose deaths involved usage of any opioid including prescription opioids, heroin, and other synthetic narcotics while 17 029 drug overdose deaths were associated with the use of prescription opioids.^2^

The rates of opioid use disorder (OUD) have increased throughout the country, with as many as 2 319 213 people reported to be abusing opioids or being dependent on them throughout the United States.^3^ The rise in OUD rates has resulted in higher utilization of treatment programs for OUD, including buprenorphine, naltrexone, and methadone. The rate of opioid-related emergency department visits increased by 106.4% from 2009 to 2014.^4^

The opioid epidemic has impacted the state of Ohio, which is among the top five states in the country with the highest rates of opioid-related overdose deaths. In 2016, there were 3613 opioid-related overdose deaths in Ohio, at a rate of 32.9 deaths per 100 000 persons, double the national rate of 13.3 deaths per 100 000.^5^ The opioid epidemic has especially impacted the city of Cincinnati, which is the third largest city in the state of Ohio. In 2016, there were 5274 reported opioid-related overdoses in the city of Cincinnati, which accounted for the most overdoses of any city in Ohio.^6^

OUD is defined as a problematic pattern of opioid use leading to clinically significant impairment or distress.^7^ While there is up-to-date data available for opioid-related overdoses and past year opioid abuse or dependency rates, there is little data available for OUD rates in different regions throughout the country. Specifically, there is a lack of available data for Ohio and the Cincinnati region. Furthermore, this analysis has been conducted to potentially identify any inequities for populations with OUD with respect to race and ethnicity status within the Cincinnati region and United States. Current evidence has suggested that inequities found in populations being treated for OUD result in significant variations in concomitant substance abuse and utilization of medication-assisted treatment.^8^ The objectives of this study were to compare the Cincinnati region to the United States with respect to the rate of treatment admissions, length of time to obtain treatment, type of treatment for OUD, and to assess if any racial inequities were observed for the aforementioned outcomes within the Cincinnati region.

## METHODS

### Data

2017 Data were obtained from the Treatment Episode Data Set: Admissions (TEDS-A), a national data system of annual admissions to substance abuse treatment facilities administered by the Substance Abuse and Mental Health Services Administration (SAMHSA).^9^ Facilities reporting TEDS-A data are those that receive state alcohol and/or drug agency funds (including Federal Block Grant funds) for the provision of substance abuse treatment. Each observation in the data represents a substance use treatment episode. If an individual seeks treatment three times in a year, they will be counted three times. The data set included records on admissions aged 12 or older, information on admission demographics (eg, age, sex, race/ethnicity, employment status) and substance abuse characteristics (eg, substances used, age at first use, route of use, frequency of use, number of prior admissions).^9^ For each episode, the primary, secondary, and tertiary substances abused were reported. SAMHSA ensures that the publicly available data complies with the Health Insurance Portability and Accountability Act.

### Statistical Analysis

All admissions with a primary substance at admission of opioids, including opioid derivatives, were included in the analysis. For the first objective, the data was stratified by the United States and Cincinnati CBSA region. For the second objective, the Cincinnati CBSA data were stratified by race: Caucasian, African American, and Other (eg, Asian, Native American).

The following outcomes were analyzed in the primary and secondary objectives: rate of treatment admissions, length of wait in days until admission, and type of treatment. Data measured on a continuous scale were summarized with means, standard deviations, ranges, and medians. Categorical data were summarized with counts and percentages. Chi-square tests were employed for categorical variables and analysis of variance for continuous variables to assess the differences between groups.

Multivariable regression models were created for each dependent study variable to isolate the effect of each outcome. Ordinal logistic regression models were used for categorical outcomes. To adjust for confounding, model covariates included observed patient and treatment facility characteristics. To assess the influence of the observed covariates, three different models were specified and performed incorporating different covariates for each outcome. The first model controlled for age and gender. The second model added the presence of any secondary and tertiary substances abused (ie, benzodiazepines, cocaine, marijuana, alcohol.) The third model added demographic and socioeconomic control variables identified in Tables 1 and 4. The three-step model specification was chosen to better understand robustness of the findings based on the observed covariates.

All analyses and graphical presentations were performed using SPSS software, version 24 (IBM, Armonk, NY). A *P* value less than 0.05 was considered statistically significant. The study was determined by the Xavier University Institutional Review Board to be exempt from their review.

## RESULTS

### Cincinnati Region versus United States

There were 678 766 US and 3298 Cincinnati region individuals admitted for OUD treatment in 2017. The patient characteristics differed significantly between the Cincinnati CBSA region and the US for OUD admissions (Table 1). The greatest difference was seen in the unemployment rate, which was 67.1% and 36.2% (*P* value < 0.0001) for the Cincinnati CBSA region and the United States, respectively. The distribution of age for the total amount of OUD admissions between the Cincinnati CBSA region and the total amount of OUD admissions for the US differed significantly depending on various age brackets in 2017.

The total percentage of OUD admissions who utilized 24-hour freestanding detox centers at the time of admission in the Cincinnati CBSA region was 29.5%, compared to 19.9% in the US (*P* value < 0.0001). The results were reversed when comparing OUD admissions who used inpatient detox centers between the Cincinnati CBSA region (0.1%) and the US (3.24%; *P* value < 0.0001). A higher percentage of OUD admissions reported having one arrest prior to admission in the Cincinnati CBSA region (9.1%) than for the US (4.7%) (*P* value < 0.0001). There was also a higher percentage of Cincinnati CBSA region OUD admissions who recorded no arrests 30 days prior to admission when compared to the United States (86.9% vs 79.2%, respectively; *P* value < 0.0001).

Differences in treatment referral sources were prevalent as well, particularly when comparing alcohol/drug use care provider and court/ criminal justice referral/DUI/DWI between both geographic regions. For the Cincinnati CBSA region, there was a lower percentage of OUD admissions that utilized alcohol/drug use care providers as a treatment referral source when compared to the US (2.7% vs 12.5%, respectively). The percentage of OUD admissions that utilized court/ criminal justice referral/DUI/DWI as their treatment referral source was 14.3% in the United States compared to 24.7% in the Cincinnati CBSA region (*P* value < 0.0001).

Statistical differences were also found throughout the Unadjusted Outcome Results by Cincinnati CBSA and United States (Table 2). For the Cincinnati CBSA region, 37.7% of OUD admissions waited 1–7 days to enter treatment compared to 12.9% of OUD admissions for the US (*P* value < 0.0001). The total percentage of OUD admissions that utilized planned medication-assisted opioid therapy also varied between the Cincinnati CBSA region and the United States. The percentage of OUD admissions that utilized planned medicationassisted opioid therapy in the Cincinnati CBSA region was 21.8%, compared to 38.6% in the US (*P* value < 0.0001). Conversely, for the Cincinnati CBSA region, 73.9% of admissions did not utilize planned medication-assisted opioid therapy, compared to 58.6% of OUD admissions in the US who did not utilize planned medication-assisted opioid therapy (*P* value < 0.0001). The rate of OUD admissions for the Cincinnati CBSA region was 1.51 per 1000 compared to the rate of admission for the US, which was 2.08 per 1000 in 2017 (*P* value < 0.0001).

The results of the regression analysis comparing the planned medication-assisted therapy and length of wait for treatment for the Cincinnati CBSA are found in Table 3a and 3b. All three model specifications found the odds of those receiving planned medication-assisted therapy to be less in the Cincinnati CBSA as compared to the US. The odds of patients receiving planned medication-assisted therapy was 0.455 (95% CI, 0.418 to 0.495; *P* value < 0.001), 0.603 (95% CI, 0.554 to 0.656; *P* value < 0.001), and 0.497 (95% CI, 0.451 to 0.546; *P* value < 0.001) for models 1, 2, and 3 respectively (Table 3a). All three models found that the odds of waiting longer for treatment in the Cincinnati CBSA were higher than the US. The odds ratios were 2.19 (95% CI, 2.06 to 2.34; *P* value < 0.001), 2.69 (95% CI, 2.53 to 2.87; *P* value < 0.001), and 2.33 (95% CI, 2.19 to 2.48; *P* value < 0.001) for models 1, 2, and 3 respectively (Table 3b). The following covariates had the most influence on the results for planned medication-assisted therapy and treatment wait time: Service Setting, Ethnicity, and Prior Arrests.

### Cincinnati Racial Inequity Analysis

In Cincinnati, there were 3102 Caucasian, 123 African American, and 73 Other admissions in 2017 (Table 4). The primary referral source for all OUD admissions in the Cincinnati CBSA region is self-referral, which represents 55.9% of admissions for all races and their recorded admissions in 2017 (*P* value < 0.0001). Differences were noted in the treatment services utilized at time of admission for OUD admissions between the three race classifications. For Caucasian admissions, 49.5% of OUD admissions utilized ambulatory non-intensive outpatient services (*P* value < 0.0001). The second highest utilized treatment service was 24-hour freestanding residential detox, which made up 28.4% of all Caucasian OUD admissions (*P* value < 0.0001). Over 54% of African American OUD admissions utilized 24-hour freestanding residential detox as their treatment service, with the second largest treatment service being ambulatory non-intensive outpatient services, representing 26.0% of all African American OUD admissions (*P* value < 0.0001). For Other OUD admissions, 46.6% utilized ambulatory non-intensive outpatient services, with the second largest treatment service being 24-hour freestanding residential detox, representing 34.2% (*P* value < 0.0001).

The age range for OUD admissions in the Cincinnati CBSA region had notable differences between admissions that were classified as Caucasian compared to all other races. For Caucasian OUD admissions, 26.5% were between 25–29 years old, being the largest percentage for that race. For African Americans, 28.5% of OUD admissions were ≥50 years old (*P* value < 0.0001). For Other, 28.8% of OUD admissions were between 30–34 years old (*P* value < 0.0001). For OUD admissions, Caucasians were older in the Cincinnati CBSA region.

There were differences in rate of treatment, utilization of planned medication-assisted opioid therapy, and days waiting for treatment among the three different race classifications (Table 5). For OUD admissions who utilized planned medication-assisted opioid therapy in the Cincinnati CBSA region, 22.4% were Caucasian, 17.1% were African American, and 5.5% were Other (*P* value < 0.0001). Similar differences were found between OUD admissions by race for number of days waiting to enter treatment between 1 to 7 days, with 37% being Caucasian, 53.7% being African American, and 41.4% being Other (*P* value = 0.0098). Rate of treatment per 1000 by race showed that 1.71 Caucasian admissions, 0.42 African American admissions, and 0.73 Other admissions were being treated per 1000 population (*P* value < 0.0001).

The results of the regression analysis comparing the planned medication-assisted therapy and length of wait for treatment by race for the Cincinnati CBSA are found in Table 6a and 6b, respectively. With respect to planned medication-assisted therapy usage, there was no statistically significant difference in the likelihood of its use between African Americans and Caucasians. However, individuals classified as Other were more likely to have planned medication-assisted therapy in two of the three model specifications. The odds ratios were 3.40 (95% CI, 1.11–10.37; *P* value = 0.032), 3.23 (95% CI 1.06–9.87; *P* value = 0.400), and 7.073 (95% 2.10–23.80; *P* value = 0.002) for models 1, 2, and 3, respectively (Table 6a).

Within the Cincinnati CBSA there was only one model that found a statistically significant difference in days waited to enter treatment. Model 1 found Other had a greater likelihood of waiting to enter treatment than African Americans (OR 1.900; 95% CI 1.09–3.31; *P* value = 0.024) (see Table 6b).

## DISCUSSION

The results of these analyses have highlighted numerous discrepancies for our three measured outcomes. First, when comparing the outcomes of the Cincinnati CBSA region and the United States, the odds of waiting longer for treatment and for not receiving planned medication-assisted therapy were significantly higher in the Cincinnati CBSA region. Second, within the Cincinnati CBSA region, those classified as Other were more likely to receive planned medication-assisted therapy compared of African Americans in two of the three model specifications. Only one model specification (Table 6A; Model 3) found that Caucasians were more likely to receive planned medicated-assisted therapy. Compared to African Americans, one model specification (Table 6B; Model 1) found those classified as Other to have a longer wait for treatment.

Our results between the treatment for OUD between the US and the Cincinnati CBSA region suggest that individuals who were admitted for OUD are not receiving the standard of care that is seen throughout the rest of the country. This is despite having one of the highest rates of needs for treatment for illicit drug use. More research and investigation can be done into the specific socioeconomic factors of all OUD admissions and how they may impact patients throughout the Cincinnati CBSA region. While we controlled for race, we did not have access to household income or specific geographic location (rural vs urban settings), which may influence our findings. It may be easier to access evidence-based treatment in a timely manner based on location and income. In addition to these socioeconomic factors, there may be a difference in specific policies and regulations.

Cincinnati has been the epicenter of the current opioid epidemic, and its treatment resources are overwhelmed in its response to the needs of those with OUD. We hypothesized that a shortage in supply may be contributing to lack of evidence-based treatment options and increased wait times to receive any treatment. Our findings suggest that the Cincinnati CBSA region does have lack of supply of treatment relative to the nation. We speculate that additional treatment resources located in the Cincinnati CBSA region may be able mitigate the opioid epidemic through faster time to treatment from identified need and increased adherence to evidence-based treatment options.

Prior studies have found multiple disparities in treatment type and time waiting to receive treatment based on race.^10^,^11^,^12^ We have found that race may play a role in receiving evidence-based treatment options within the Cincinnati CBSA region. However, this finding was not robust to our alternative model specifications. We suspect that this finding was likely due to longstanding cultural and socioeconomic issues such as insurance type.^13^,^14^ While there may be racial inequity in the specific type of treatment, we did not find any racial inequity in the length of time waiting for treatment. It is possible that localized treatment options available may drive the type of treatment. However, we do not want to minimize the role of culture or socioeconomic status.

### Limitations

Limitations of our research include the dependability and accessibility of the TEDS-A data. Data in TEDS-A is dependent on public fund availability, which varies by state, and may result in missing data sets for each state that reported to TEDS-A in 2017. Most of the data in TEDS-A is also self-reported, with no other forms of validation available throughout the reporting process. Individual facilities are required to electronically report the data to their state as a requirement of receiving federal funds. The state level data is then reported to SAMHSA. The data did not capture admissions to the Veteran’s Health Administration or 100% private institutions, which may impact our results. Facility-level information such as ownership, size, and provider to patient ratio was not included in the data set. Finally, there may selection bias and unobserved cofounding covariates not included in the data. As a result of these limitations, the results of this observational study should be interpreted cautiously.

## CONCLUSION

Our research suggests that there is a difference between the treatment capacity of the Cincinnati CBSA region and the rest of the US for 2017. The higher percentage of OUD admissions that spent 1–7 days waiting to enter treatment suggests that the Cincinnati CBSA region does not have the same capacity to treat individuals with OUD when compared to the rest of the United States. There were also higher percentages of OUD admissions in the Cincinnati CBSA area that did not use planned medication-assisted opioid therapy when compared to the rest of the United States. Increasing the supply of treatment services may reduce the deleterious effects of the opioid epidemic in the Cincinnati CBSA.

## Figures and Tables

**Table 1:** Patient Characteristics by Cincinnati CBSA and the United States

	United States	Cincinnati CBSA	*P* Value
**N**	678	776	3298
**Age**			
<18	0.2%	0.1%	
18–24	11.8%	10.5%	
25–29	23.2%	26.1%	
30–34	19.9%	23.3%	
35–39	14.0%	17.6%	
40–44	8.2%	8.7%	
45–49	7.7%	6.3%	
≥50	15.0%	7.5%	<0.0001
**Gender**			
Female	38.7%	47.5%	
Male	61.3%	52.5%	
Unknown	0.0%	0.0%	<0.0001
**Race**			
African American	13.3%	3.7%	
Caucasian	72.7%	94.1%	
Asian	0.5%	0.0%	
Other	13.5%	2.2%	<0.0001
**Ethnicity**			
Hispanic or Latino	12.4%	0.9%	
Not Hispanic or Latino	84.8%	98.5%	<0.0001
**Married**			
Yes	5.3%	10.7%	
No	67.5%	89.1%	
Unknown	23.2%	0.2%	<0.0001
**Education**			
No high school diploma	24.6%	24.9%	
High school diploma	48.6%	54.9%	
Attends/graduated college	22.5%	19.9%	
Unknown	4.3%	0.4%	<0.0001
**Employment**			
Full-time	12.0%	12.1%	
Part-time	5.7%	6.9%	
Unemployed	36.2%	67.1%	
Unknown/other	46.1%	13.9%	<0.0001
**Number of Arrests 30 Days Prior to Admission**			
None	79.2%	86.9%	
One	4.7%	9.1%	
Two or more	1.0%	0.7%	
Unknown	15.1%	3.3%	
**Service Setting at Admission**			
Detox, 24-hour, hospital inpatient	3.2%	0.1%	
Detox, 24-hour, freestanding residential	19.9%	29.5%	
Rehab/residential, hospital (non-detox)	0.2%	0.0%	
Rehab/residential, short term (30 days or fewer)	8.9%	0.7%	
Rehab/residential, long term (more than 30 days)	6.8%	5.9%	
Ambulatory, intensive outpatient	10.4%	7.8%	
Ambulatory, non-intensive outpatient	48.6%	48.6%	
Ambulatory, detoxification	2.0%	7.4%	<0.0001
**Treatment Referral Source**			
Individual (self-referral)	57.0%	55.9%	
Alcohol/drug use care provider	12.5%	2.7%	
Other health care provider	4.8%	3.1%	
School	0.0%	0.0%	
Employer/EAP	0.2%	0.1%	
Other community referral	8.6%	10.7%	
Court/criminal justice referral/DUI/DWI	14.3%	24.7%	
Unknown	2.6%	2.9%	<0.0001

**Table 2:** Unadjusted Outcome Results by Cincinnati CBSA and the United States

	United States	Cincinnati CBSA	*P* Value
**N** 678 776	3298		
**Planned Medication-Assisted Opioid Therapy**			
Yes	38.6%	21.8%	
No	58.6%	73.9%	
Unknown	2.8%	4.3%	<0.0001
**Number of Days Waiting to Enter Treatment**			
0	45.5%	32.9%	58.4
1–7	12.9%	37.7%	70.6
8–14	2.4%	1.6%	
15–30	1.7%	0.9%	
31+	1.0%	0.9%	
Unknown	36.5%	26.0%	<0.0001
**Rate of Treatment Admissions from General Population, per 1000**	2.08	1.51	<0.0001

**Table 3:** Regression Results by Cincinnati CBSA and the United States: (A) Planned Medication-Assisted Opioid Therapy (MAT), (B) Number of Days Waiting to Enter Treatment^a^

**(A) Planned MAT**	**Model 1**			
	**OR**	**95% CI**		***P* Value**
	0.455	0.418	0.495	<0.000
	**Model 2**			
	0.603	0.554	0.656	<0.000
	**Model 3**			
	0.497	0.451	0.546	<0.000
**(B) Number of Days Waiting**	**Model 1**			
	**OR**	**95% CI**		***P* Value**
	2.197	2.062	2.341	<0.000
	**Model 2**			
	2.697	2.532	2.875	<0.000
	**Model 3**			
	2.335	2.191	2.489	<0.000

a Model 1 includes Age and Gender covariates; Model 2 adds second and third substance abused; Model 3 adds Race, Ethnicity, Married, Education, Employment, Prior Arrests, Service Setting, and Referral Source covariates.

**Table 4:** Patient Characteristics by Race/Ethnicity for Cincinnati CBSA

	Caucasian	African American	Other	*P* Value
**N**	3102	123	73	
**Age**				
<18	0.1%	0.0%	0.0%	
18–24	10.6%	7.3%	9.6%	
25–29	26.5%	16.3%	27.4%	
30–34	23.2%	21.1%	28.8%	
35–39	17.8%	12.2%	19.2%	
40–44	8.7%	8.9%	6.8%	
45–49	6.4%	5.7%	5.5%	
≥50	6.8%	28.5%	2.7%	<0.0001
**Gender**				
Female	48.3%	37.4%	34.2%	
Male	51.7%	62.6%	65.8%	
Unknown	0.0%	0.0%	0.0%	0.0099
**Ethnicity**				
Hispanic or Latino	0.7%	0.8%	12.3%	
Not Hispanic or Latino	98.8%	99.2%	84.9%	<0.0001
**Married**				
Yes	10.8%	8.1%	9.6%	
No	89.0%	91.9%	89.0%	
Unknown	0.1%	0.0%	1.4%	0.1934
**Education**				
No high school diploma	24.5%	35.8%	21.9%	
High school diploma	55.0%	49.6%	60.3%	
Attends/graduated college	20.2%	14.6%	15.1%	
Unknown	0.3%	0.0%	2.7%	0.0016
**Employment**				
Full-time	12.5%	7.3%	2.7%	
Part-time	7.0%	4.9%	6.8%	
Unemployed	66.7%	73.2%	72.6%	
Unknown/other	13.7%	14.6%	17.8%	0.0545
**Number of Arrests 30 Days Prior to Admission**				
None	86.8%	90.2%	82.2%	
One	9.1%	8.9%	9.6%	
Two or more	0.7%	0.0%	2.7%	
Unknown	3.4%	0.8%	5.5%	<0.0001
**Service Setting at Admission**				
Detox, 24-hour, hospital inpatient	0.1%	0.0%	0.0%	
Detox, 24-hour, freestanding residential	28.4%	54.5%	34.2%	
Rehab/residential, hospital (non-detox)	0.0%	0.0%	0.0%	
Rehab/residential, short term (30 days or fewer)	0.6%	0.0%	2.7%	
Rehab/residential, long term (more than 30 days)	5.8%	8.9%	5.5%	
Ambulatory, intensive outpatient	7.7%	8.9%	9.6%	
Ambulatory, non-intensive outpatient	49.5%	26.0%	46.6%	
Ambulatory, detoxification	7.8%	1.6%	1.4%	<0.0001
**Treatment Referral Source**				
Individual (self-referral)	55.0%	73.2%	63.0%	
Alcohol/drug use care provider	2.8%	0.8%	0.0%	
Other health care provider	3.1%	0.8%	4.1%	
School	0.0%	0.0%	0.0%	
Employer/EAP	0.1%	0.8%	0.0%	
Other community referral	10.6%	12.2%	11.0%	
Court/criminal justice referral/DUI/DWI	25.5%	9.8%	17.8%	
Unknown	2.9%	2.4%	4.1%	0.0030

**Table 5:** Unadjusted Outcome Results by Race/Ethnicity in Cincinnati CBSA

	Caucasian	African American	Other	*P* Value
**N**	3102	123	73	
**Planned Medication-Assisted Opioid Therapy**				
Yes	22.4%	17.1%	5.5%	
No	73.3%	79.7%	89.0%	
Unknown	4.4%	3.3%	5.5%	<0.0001
**Number of Days Waiting to Enter Treatment**				
0	32.8%	35.8%	30.1%	
1–7	37.0%	53.7%	41.1%	
8–14	1.6%	1.6%	0.0%	
15–30	1.0%	0.0%	1.4%	
31+	0.9%	0.8%	0.0%	
Unknown	26.7%	8.1%	27.4%	0.0098
**Rate of Treatment Admissions from General Population, per 1000**	1.71	0.42	0.73	<0.0001

**Table 6:** Regression Results in Cincinnati CBSA by Race: (A) Planned Medication-Assisted Opioid Therapy (MAT), (B) Number of Days Waiting to Enter Treatment^a^

**(A) Planned MAT**			**Model 1**		
	**Race**	**OR**	**95% CI**		***P* Value**
	African American	Ref			
	Caucasian	0.715	0.442	1.160	0.175
	Other	3.396	1.112	10.374	0.032
			**Model 2**		
	African American	Ref			
	Caucasian	0.709	0.437	1.149	0.162
	Other	3.228	1.056	9.870	0.400
	**Model 3**				
	African American	Ref			
	Caucasian	1.898	1.032	3.489	0.039
	Other	7.073	2.101	23.804	0.002
**(B) Number of Days Waiting Model 1**	**Race**	**OR**	**95% CI**		***P* Value**
	African American	Ref			
	Caucasian	1.001	0.648	1.561	0.979
	Other	1.900	1.089	3.310	0.024

			**Model 2**		
	African American	Ref			
	Caucasian	0.859	0.544	1.356	0.513
	Other	1.600	0.901	2.842	0.109
			**Model 3**		
	African American	Ref			
	Caucasian	1.064	0.586	1.932	0.838
	Other	1.289	0.609	2.728	0.507

a Model 1 includes Age and Gender covariates; Model 2 adds second and third substance abused; Model 3 adds Ethnicity, Married, Education, Employment, Prior Arrests, Service Setting, and Referral Source covariates.
